# Modified Shen Ling Bai Zhu San Combined with Its Essential Oil Regulates Growth Performance, Lipid Metabolism and Intestinal Health in Silky Fowls

**DOI:** 10.3390/ani16152333

**Published:** 2026-07-30

**Authors:** Xiujun Wang, Xinglin Gao, Yimu Ma, Yun Gao, Shuhong Chai, Menghui Cao, Jieyi Huang, Weijie Lv, Qian Qu, Shining Guo

**Affiliations:** 1 College of Veterinary Medicine, Henan University of Animal Husbandry and Economy, No. 6 Wenyuan Road, Zhengzhou 450046, China; wxjfly@126.com; 2 College of Veterinary Medicine, South China Agricultural University, Guangzhou 510642, China; 20241029002@stu.scau.edu.cn (X.G.); ym_ma@stu.scau.edu.cn (Y.M.); gaoyun@stu.scau.edu.cn (Y.G.); shchai@stu.scau.edu.cn (S.C.); caomenghui@stu.scau.edu.cn (M.C.); 15802033344@163.com (J.H.); lvwj@scau.edu.cn (W.L.); 3 College of Animal Science, Anhui Science and Technology University, Chuzhou 233100, China; 4 Guangdong Technology Research Center for Traditional Chinese Veterinary Medicine and Nature Medicine, Guangzhou 510642, China

**Keywords:** silky fowls, modified Shen Ling Bai Zhu San, essential oil, lipid metabolism, gut microbiota

## Abstract

Background: Antibiotic exposure disrupts gut microbiota and lipid metabolism, hindering healthy poultry farming, especially for medicinal Silky fowls. Given that essential oils are prone to volatilization during the decoction of traditional Chinese medicine, this study evaluated modified Shen Ling Bai Zhu San and its essential oils (SLEO) on growth, lipid metabolism, and intestinal health in Silky fowls. Methods: 540 Silky fowls were divided into CON, ATB, ATB+SLEO, and SLEO groups. The cecal microbiota was analyzed via 16S rRNA sequencing. Results: ATB improved weight gain but increased abdominal fat, liver lipid accumulation, and serum triglycerides/cholesterol. Lipid transporter and metabolism gene expression was upregulated, while CPT1A and PPARα were downregulated. SLEO alone significantly improved intestinal villus morphology, as evidenced by a higher villus height/crypt depth (V/C) ratio compared to the ATB group (*p* < 0.05), along with reduced mortality and enhanced barrier gene expression, without adverse lipid effects. Antibiotics reduced cecal microbial α/β diversity, which SLEO reversed. Enriched SLEO-associated strains correlated strongly with lipid metabolism. Conclusion: SLEO may serve as an antibiotic alternative, regulating lipid metabolism, reducing fat deposition, maintaining gut microbiota homeostasis, and alleviating antibiotic-induced adverse effects.

## Simple Summary

Antibiotics are commonly used in poultry farming to keep chickens healthy, but they can cause long-term negative side effects, especially disrupting the beneficial gut microbial community and lipid metabolism. We tested whether modified Shen Ling Bai Zhu San and its essential oils reduce the damage caused by antibiotics and improve fat processing in Silky fowl chickens. We found that even though antibiotics were only given in the first 10 days of life, they still reduced the variety of beneficial gut bacteria and increased fat accumulation by 75 days of age. Supplementation with modified Shen Ling Bai Zhu San and its essential oils restored the diversity of gut bacteria and promoted beneficial microbes involved in sugar and fat regulation. These beneficial bacteria were strongly linked to healthier fat levels and improved expression of fat-processing genes. Our findings demonstrate that modified Shen Ling Bai Zhu San and its essential oils enhance growth, balance fat metabolism, restore gut health, and reduce metabolic damage from early antibiotic exposure. This work helps support the development of healthy, antibiotic-free poultry farming, which provides safer, healthier poultry products for the public.

## 1. Introduction

Antibiotics have been used to treat bacterial infections since their discovery and have saved millions of lives in the battle against microbes [1]. For over six decades, they have been employed in some countries and regions as feed additives to promote growth in livestock and poultry [2]. The intensification of farming practices has inevitably led to antibiotic overuse. While subtherapeutic doses of antibiotics can enhance growth performance, prevent disease, and reduce inflammation, they also result in antibiotic residues, an increase in antimicrobial resistance genes, and disruption of the gut microbiota [3]. A growing body of evidence further indicates that chronic exposure to low-dose antibiotics promotes adipose tissue deposition [4]. In broiler studies, antibiotics have been shown to significantly increase abdominal fat accumulation [5]. Excessive abdominal fat deposition can induce dyslipidemia, impair reproductive performance, and lead to a range of adverse consequences [4]. Pharmacokinetic studies have shown that a number of antibiotics remain in the organs, muscles, and skin of Silky fowls for a longer period of time. For example, after the administration of enrofloxacin, Silky fowls need a withdrawal period of 284 days to metabolize the residual antibiotics [6]. Another study on Silky fowls also showed that trimethoprim was present in significantly higher concentrations in the muscle of Silky fowls and remained in their tissues for a longer period of time than in 817 broilers [7]. Therefore, the livestock industry is urgently looking for more suitable and potential alternatives to antibiotics for the healthy breeding of Silky fowls. However, the effects of antibiotic alternatives on lipid metabolism and intestinal health in indigenous chicken breeds remain largely unknown.

In recent years, herbs, probiotics, and other alternatives have been continuously explored for their potential to replace antibiotics in promoting growth and preventing diseases [8,9]. Studies have shown that many herbal formulations not only enhance the growth of broiler chickens but also help prevent bacterial diseases. Our previous research has demonstrated that a Chinese herbal mixture improves growth performance, enhances antioxidant and anti-inflammatory responses, regulates immune function, and maintains the homeostasis of the gut microbiota in both laying hens and broilers [10,11]. Moreover, in an *E. coli*-induced model of acute bacterial infection in chickens, the herbal mixture was found to alleviate liver and intestinal damage [12]. Shen Ling Bai Zhu San (SLBZS), a traditional Chinese medicine, was formulated in the Song Dynasty and documented in the Taiping Huimin Heji Ju Fang [13]. Recent studies have found that SLBZS regulate the gut microbiota, strengthen the intestinal barrier, reduce the level of pro-inflammatory factors, and improve the immune function and antioxidant capacity [14,15]. In our previous studies, SLBZS was shown to alleviate colitis, antibiotic-induced diarrhea, and weaning stress in piglets by modulating the gut microbiota, intestinal barrier function, and intestinal immunity [16,17,18]. Essential oils are a distinct and highly bioactive class of compounds in traditional Chinese medicine (TCM). These volatile, plant-derived oils are obtained through steam distillation, which also makes them prone to loss during herbal processing. Nevertheless, they exhibit significant biological activity. Numerous studies have shown that the essential oils of many plants play important roles in antioxidant and antimicrobial activities [19]. Thymol and carvacrol, have been reported to reduce oxidative stress, alleviate weight gain, and influence the colonization of gut microbiota [20].

Silky fowls are a local Chinese breed known for their nourishing properties but are difficult to breed. Silky fowls possess higher medicinal and dietary value compared to ordinary chickens [21]. They are a key component in the well-known Chinese herbal formula “Wu Ji Bai Feng Wan,” which is renowned for nourishing qi and blood [22]. Therefore, based on previous experiments and considering the volatile nature of herbal components, this study explores the effects of SLEO (a composite mixture of SLBZS powder and additionally extracted essential oils) on the growth performance and intestinal health of Silky fowls.

In this study, we employed a multi-target approach to examine the effects of antibiotics and SLEO on growth performance, nutrient transport, lipid metabolism, and intestinal barrier function in Black-boned chickens. We further compared the structural alterations of the intestinal microbiota induced by antibiotics versus SLEO. Correlation analyses were conducted to explore the interactions within the gut-liver axis and their associations with gut microorganisms. The findings aim to provide additional evidence that TCM can serve not only as an effective alternative to antibiotics, but also as a means to alleviate the adverse effects associated with antibiotic use. The classic SLBZS is a powdered traditional Chinese medicine formula. However, its volatile oil components—which possess antibacterial and gut-regulating properties—are highly volatile and are lost in large quantities during conventional decoction or direct ingestion. To overcome this limitation, we prepared a modified SLEO by extracting essential oils from the herbal mixture and reincorporating them into the powdered formulation at a 10:1 ratio. This method aims to preserve all of the formulation’s bioactive components and maximize its potential effects on the gut microbiota and host metabolism.

## 2. Materials and Methods

### 2.1. Preparation of Modified Shen Ling Bai Zhu San Combined with Essential Oil

The medicinal materials used, including Ginseng (6.9%), *Poria cocos* (6.9%), *Atractylodes macrocephala* (6.9%), *Glycyrrhiza uralensis* (6.9%), *Dioscorea opposita* (6.9%), *Lablab purpureus* (5.17%), *Semen nelumbinis* (3.45%), *Platycodon grandiflorum* (3.45%), *Coix lacryma-jobi* (3.45%), Furnace Soil (10%), *Sophora flavescens* (10%), *Portulaca oleracea* (10%), *Thalictrum glandulosissimum* (10%), and Plant Soot (10%) were all purchased from Hebei Anguo Qi’an Pharmaceutical Co., Ltd. (Baoding, China). The essential oil was provided by Guangdong Weilai Biotechnology Co., Ltd. (Guangzhou, China). Its main components include active ingredients in plant essential oils such as thymol (derived from Ginseng and *Dioscorea opposita*), carvacrol (Ginseng, *Dioscorea opposita*, and *Glycyrrhiza uralensis*), and cinnamaldehyde (*Poria cocos* and *Glycyrrhiza uralensis*). SLEO is formed by mixing powdered SLBZS and essential oil in a 10:1 ratio. The proximate chemical composition (crude protein, crude fat, crude fiber, ash, total phosphorus, and calcium) of SLEO was measured according to AOAC (Association of Official Analytical Chemists) methods (AOAC, 2010) [23]. The polysaccharide, polyphenol, and flavonoid contents and approximate chemical composition of SLEO were shown in Table S1.

### 2.2. Preparation and Administration of Antibiotics

All antibiotics were purchased from Guangdong Wens Dahuanong Biotechnology Co., Ltd. (Yunfu, China), including Compound Amoxicillin, Florfenicol, Tylvalosin, and Gentamicin. All the veterinary antibiotics (ATB) used in this study were selected based on the original clinical breeding protocol and used according to the manufacturer’s instructions. We used Compound Amoxicillin (1 g/kg) in the morning of days 1 to 4, Florfenicol (0.3 g/kg) and Tylvalosin (1 g/kg) in the afternoon of days 1 to 5, and Compound Amoxicillin (1 g/kg) and Gentamicin (2 g/kg) on days 8 to 10.

### 2.3. Experimental Animals and Diets

The 1-day-old Silky fowls were supplied by Wen’s Food Group Co., Ltd. (Yunfu, China). As a designated clinical practice base for experimental animals of South China Agricultural University, Wen’s Poultry Farm provided an optimal site for conducting the clinical practice component of this study. All procedures of animal experiments in this study have been approved by the Animal Ethics Committee of the South China Agricultural University (2024F365). Based on a feeding density of 11 birds/m^2^, 540 Silky fowls were randomly assigned to 4 groups, each with 4 replicates (30 or 35 birds per replicate). Control (CON) group fed a basal diet (Table S2), antibiotics (ATB) group watered with antibiotics (from day 1 to day 10) and fed a basal diet, SLEO group fed a basal diet supplemented with 2.2 g/kg SLEO (from day 1 to day 75), and ATB+SLEO group watered with antibiotics (from day 1 to day 10) and fed a basal diet supplemented with 2.2 g/kg SLEO (from day 11 to day 75).

### 2.4. Sample Collection

At the end of the 75th day of the experiment, samples were collected after a 12 h fast. Five 75-day-old Silky fowls from each group were randomly selected and weighed. The average body weight (ABW) of each replicate was recorded. The blood was collected and centrifuged at 3000 r/min for 5 min, then the upper serum layer was separated and stored at −80 °C. The liver and small intestine were immediately collected, and the liver was fixed in 4% paraformaldehyde and the small intestine fixed in Carnot’s solution. Meanwhile, duodenal digestive fluid, jejunal mucosa, jejunum and ileum tissue and abdominal fat were collected and quickly frozen at −80 °C for further analysis. The contents of the cecum were collected aseptically and then stored at −80 °C for 16S rRNA analysis.

### 2.5. Histology of the Liver

The frozen liver sections of Silky fowls were stained with oil red O solution, and the images were acquired for analysis at 400× visual field. The intestinal morphology of the jejunum and ileum of each group was examined by AB-PAS staining. The villus height (VH) and crypt depth (CD) of the jejunum and ileum were compared at 40× visual field using ToupView (version 4.12) micrographic image measurement analysis software. The specific steps for AB-PAS staining are listed in Table S3.

### 2.6. Biochemical Tests and Enzyme-Linked Immunosorbent Assays (ELISA)

Serum levels of Growth Hormone (GH), Estradiol (E2) and Follicle-Stimulating Hormone (FSH) were measured by ELISA kit, which was purchased from Shanghai Mlbio Co., Ltd. (Shanghai, China). The concentration of Amylase, Trypsin, and Lipase in the duodenal digestive fluid was determined using ELISA kits, which were purchased from Jiangsu Meimian Industrial Co., Ltd. (Yancheng, China). Serum levels of triglycerides (TG), total cholesterol (TC), Low-density Lipoprotein Cholesterol (LDL-C) and High-density Lipoprotein Cholesterol (HDL-C) were measured by assay kit, which was purchased from Nanjing Jiancheng Bioengineering Institute (Nanjing, China). All measurements were performed in accordance with the manufacturer’s instructions.

### 2.7. Quantitative Real-Time PCR Analysis

Total tissue RNA was extracted using the RNA isolater Total RNA Extraction Reagent kit, and RNA was reverse transcribed to cDNA using the HiScript III RT SuperMix for qPCR (+gDNA wiper) reverse transcription kit. The reaction system was configured and performed according to the ChamQ Universal SYBR qPCR Master Mix kit, and the relative expression of target genes was analyzed by the 2^−∆∆Ct^ method. These kits were purchased from Nanjing Vazyme Biotech Co., Ltd. (Nanjing, China). The primers were synthesized by Tsingke Biotechnology Co., Ltd. (Beijing, China). The primer sequences are listed in Table S4.

### 2.8. 16S rRNA Sequencing Analysis

DNA extraction from fecal samples was performed using the DNeasy PowerSoil Kit (Mo Bio/QIAGEN, Hilden, Germany) according to the manufacturer’s instructions. The absorbance values of DNA at 260/280 nm were measured using a fluorescence spectrophotometer to assess the concentration of sample DNA. The quality of DNA was detected by 1% agarose gel electrophoresis. Primer sequences were F: ACTCCTACGGGAGGCAGCA and R: GGACTACHVGGGTWTCTAAT. The V3–V4 region of the microbial 16S rRNA gene was amplified by PCR. Sequencing was performed by Shanghai Personal Biotechnology Co., Ltd. (Shanghai, China) using the Illumina MiSeq gene sequencing platform. In summary, total DNA was extracted from cecal contents using the DNeasy PowerSoil kit, and the DNA concentration and quality were assessed by spectrophotometry and agarose gel electrophoresis. The V3–V4 regions of the 16S rRNA gene were amplified using primers 338F and 806R, and paired-end sequencing was performed on the Illumina MiSeq platform. On the Paisenno Gene Cloud platform, the sequencing data were processed using QIIME2 (April 2019 version) and R (version 3.6.1) packages (ggplot2 (version 3.3.5), ape (version 5.3), pheatmap (version 1.0.12), and VennDiagram) (version 1.6.20) to analyze microbial diversity and composition.

### 2.9. Statistical Analysis

All data were presented as mean ± SD with *n* ≥ 4. Statistical analysis of the experimental data was performed using IBM SPSS Statistics 22 software. After confirming normality and homogeneity of variance, differences among multiple groups were assessed by one-way analysis of variance or the non-parametric Kruskal–Wallis test. Graphical representations were generated with GraphPad Prism 9.5 software. A *p*-value < 0.05 was considered statistically significant.

## 3. Results

### 3.1. Effect of SLEO on Growth Performance and Lipid Deposition of Silky Fowls

**Figure 1:**
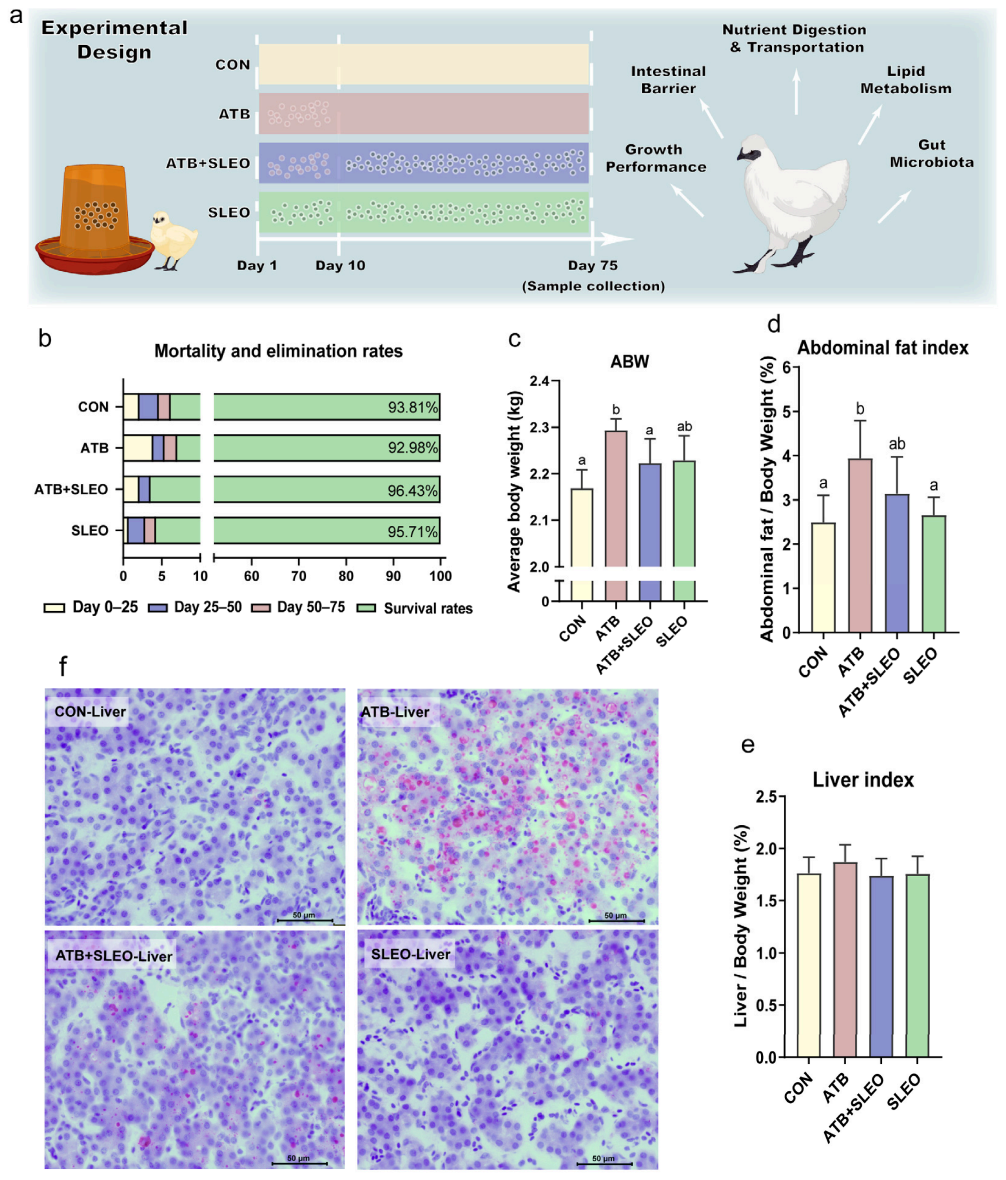
Effect of SLEO on growth performance and lipid deposition of Silky fowls supplemented with antibiotics. (**a**) Experimental design; (**b**) mortality and elimination rates; (**c**) average body weight; (**d**) abdominal fat index; (**e**) liver index; (**f**) representative oil red O staining of liver tissue sections from each group. Scale bar: 50 μm. Different letters indicate significant differences between groups (*p* < 0.05).

According to the design of the animal experimental protocol (Figure 1a), we monitored the survival rate of Silky fowls in each group at each growth stage (Figure 1b). Among the four groups, the ATB group had the lowest survival rate at the end of the experiment, at 92.98%. The survival rates of the ATB+SLEO and SLEO groups were 95.71% and 96.43%, respectively, higher than the 93.81% of the CON group. Compared with the CON group, both the ATB group and SLEO group showed an increase in ABW. However, the effect of SLEO was less pronounced than that of ATB, and the difference was not statistically significant (Figure 1c). It is noteworthy that ATB significantly increased abdominal fat weight, an effect that was significantly mitigated by SLEO intervention (Figure 1d). Importantly, the weight gain induced by SLEO was not accompanied by an increase in abdominal fat in Silky fowls. Although the liver index showed a non-significant elevation in the ATB group (Figure 1e), we observed pronounced lipid droplet deposition in the livers of ATB-treated chickens. In contrast, only a small number of lipid droplets were present in the ATB+SLEO group, while none were detected in the CON or SLEO groups (Figure 1f). These results demonstrated that the growth-promoting effect of antibiotics promotes abnormal fat deposition, particularly in visceral adipose tissue.

### 3.2. Effect of SLEO on Blood Lipids, Digestive Enzymes, and Reproductive Hormones

**Figure 2:**
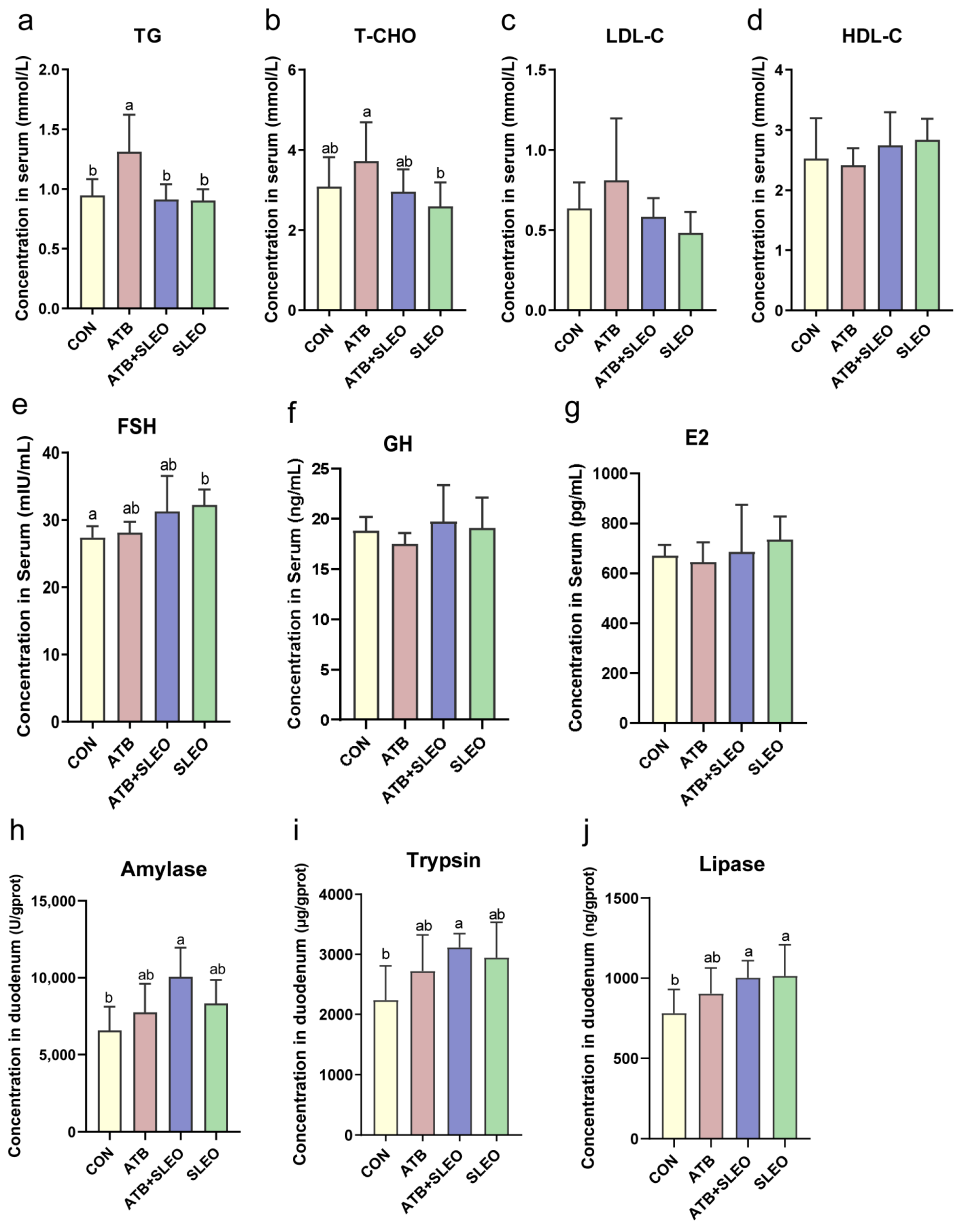
Effect of SLEO on blood lipids, digestive enzymes, and reproductive hormones. (**a**–**g**) The level of TC, TG, LDL-C, HDL-C, FSH, GH, and E2 in the serum of Silky fowls; (**h**–**j**) the concentration of amylase, trypsin, and lipase in the duodenal digestive fluid of Silky fowls. Different letters indicate significant differences between groups (*p* < 0.05).

Excessive fat accumulation, particularly visceral fat deposition, serves as a key driver of dyslipidemia [5]. Dyslipidemia, in turn, can lead to reproductive dysfunction and other related issues, while digestive function is closely intertwined with this cascade of metabolic disturbances [24]. Therefore, serum lipid profiles, reproductive hormones, and digestive enzymes in the duodenum were measured using ELISA. Compared to the CON group, the ATB group significantly increased the level of serum TG in Silky fowls (Figure 2a). In comparison with the SLEO group, the ATB group significantly elevated the level of T-CHO (Figure 2b). Furthermore, relative to the other three groups, the ATB group exhibited a trend of increased LDL-C and decreased HDL-C (Figure 2c,d), though the differences were not significant. In the ATB+SLEO group, the levels of all four lipid parameters showed no significant differences from those in either the CON group or the SLEO group. Compared to the CON group, antibiotic treatment reduced serum levels of GH and E2, while in the ATB+SLEO and SLEO groups, their levels increased, though these changes were not significant (Figure 2f,g). The serum FSH level in the SLEO group was significantly higher than that in the CON group, whereas the combination with antibiotics attenuated the promoting effect of SLEO on FSH (Figure 2e). Antibiotics increased the content of digestive enzymes (amylase, trypsin, and lipase) in the duodenal fluid, but the changes were not significant (Figure 2h–j). The ATB+SLEO group markedly promoted the levels of amylase, trypsin, and lipase, while SLEO specifically enhanced the content of lipase (Figure 2h–j). These findings suggested that while antibiotics induced dyslipidemia and altered hormone levels in Silky fowls, the supplementation with SLEO could mitigate these adverse effects and further enhance digestive enzyme activity.

### 3.3. Effect of SLEO on Lipid Metabolism in Silky Fowls

**Figure 3:**
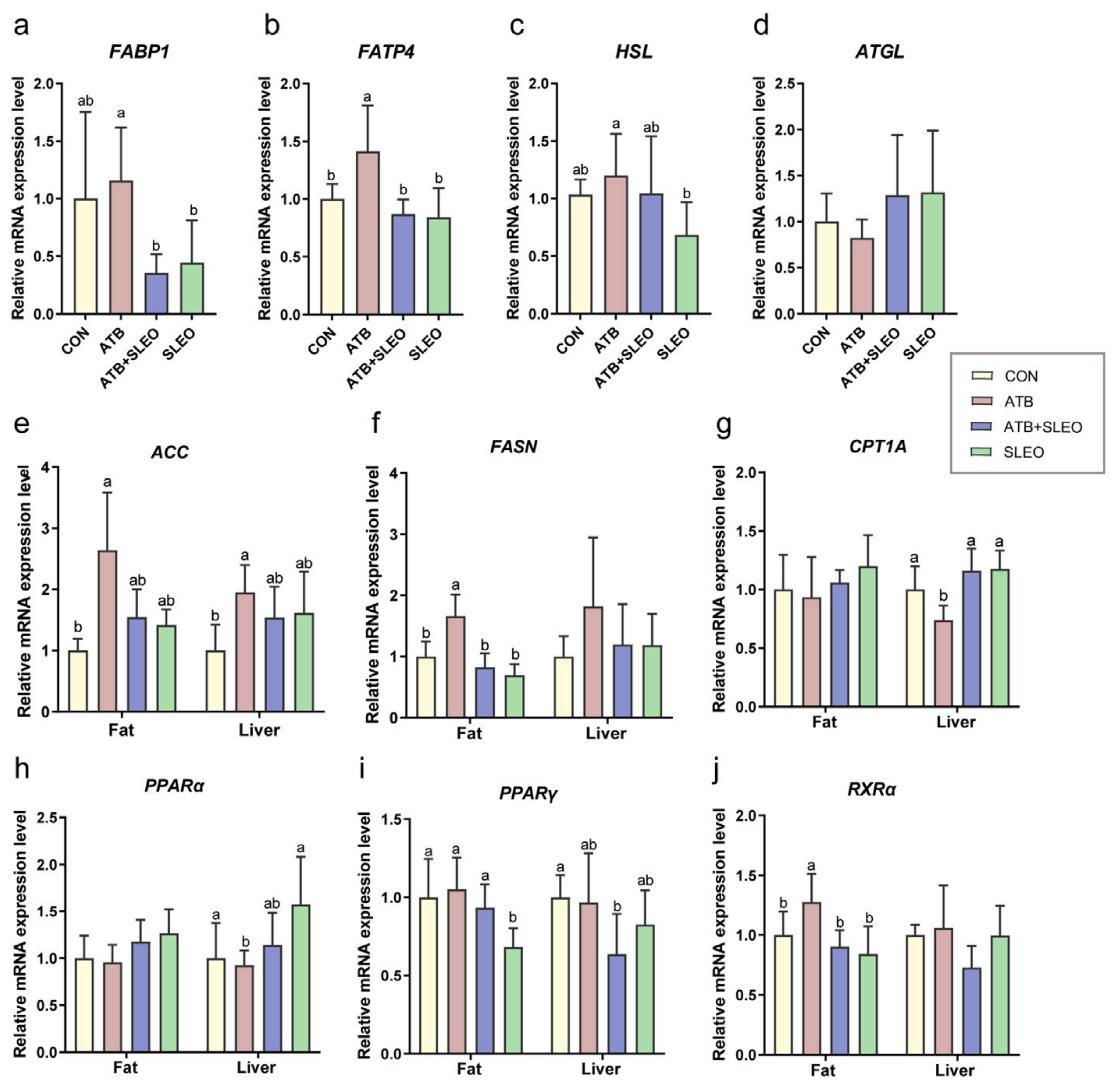
Effect of SLEO on lipid metabolism in Silky fowls supplemented with antibiotics. (**a**–**d**) The relative mRNA expression of FABP1, FATP4, HSL, ATGL in the abdominal fat of Silky fowls. (**e**–**g**) The relative mRNA expression of ACC, FASN, CPT1A in the abdominal fat and liver of Silky fowls. (**h**–**j**) The relative mRNA expression of PPARα, PPARγ, RXRα in the abdominal fat and liver of Silky fowls. Different letters indicate significant differences between groups (*p* <0.05).

Subsequently, to investigate the regulatory effect of SLEO on antibiotic-induced dyslipidemia, we analyzed lipid metabolism-related genes in the abdominal fat and liver of Silky fowls. The lipid transport proteins FABP1 and FATP4 were lower in the ATB+SLEO and SLEO groups compared with the ATB group (Figure 3a,b). The Hormone-Sensitive Lipase (HSL) was increased after the intervention of ATB, and SLEO had a down-regulation of HSL compared with ATB (Figure 3c). However, there was only a tendency for adipose triglyceride lipase (ATGL) to be elevated after SELO intervention (Figure 3d). Relative mRNA expression of Acetyl-CoA carboxylase (ACC) and fatty acid synthase (FASN) was increased in liver and adipose tissue in the ATB group compared with CON (Figure 3e,f). In contrast, the expression of ACC and FASN in the ATB+SLEO and SLEO groups did not differ from that in the CON group. Expression of the fatty acid β-oxidation rate-limiting enzyme, Carnitine palmitoyltransferase 1A (CPT1A), was down-regulated in ATB Silky fowls, and it showed a consistent up-regulation in both liver and fat in the ATB+SLEO and SLEO groups (Figure 3g). In the peroxisome proliferator-activated receptor (PPAR) signaling pathway, the expression of PPARα in the liver of the ATB group was reduced by ATB compared with the CON and SLEO groups, and PPARγ in the fat of SLEO Silky fowls was significantly lower than that of the other three groups (Figure 3h,i). The expression of Retinoid X receptor α (RXRα) in the fat and liver of the ATB group was higher than in the other three groups (Figure 3j). Collectively, these data suggest that SLEO can regulate lipid transport, synthesis, lipolysis, and fatty acid β-oxidation in Silky fowls, and that this may be associated with the PPAR signaling.

### 3.4. Effect of SLEO on Nutrient Digestion and Transport of Silky Fowls

**Figure 4:**
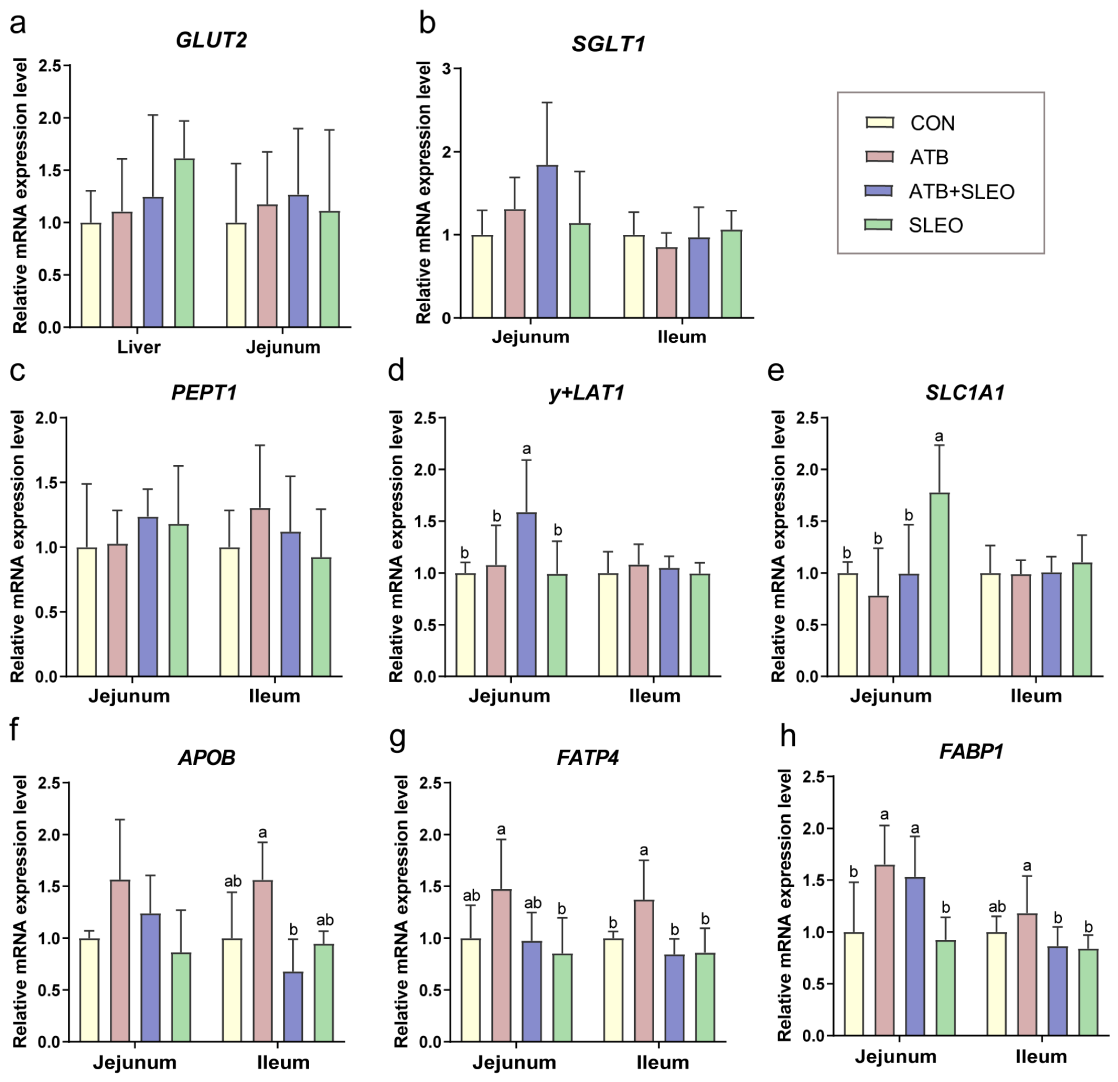
Effect of SLEO on nutrient digestion and transport in Silky fowls supplemented with antibiotics. The relative mRNA expression of (**a**) GLUT2 and (**b**) SGLT1 in the jejunum and ileum; (**c**–**e**) the relative mRNA expression of PEPT1, y+LAT, and SLC1A1 in the jejunum and ileum; (**f**–**h**) the relative mRNA expression of APOB, FATP4, and FABP1 in the jejunum and ileum. Different letters indicate significant differences between groups (*p* < 0.05).

Furthermore, we further investigated the effects of SLEO on the digestion and transport processes of nutrients in the jejunum and ileum of Silky fowls. No significant differences were found in the glucose transporter 2 (GLUT2), sodium-dependent glucose transporter 1 (SGLT1) and proton-coupled peptide transporter type 1 (PEPT1) among all groups of Silky fowls (Figure 4a–c). In the jejunum, the relative mRNA expression of the y+L amino acid transporter 1 (y+LAT1) was significantly higher in the ATB+SLEO group than that in the other groups, and the relative mRNA expression of solute carrier family 1 member 1 (SLC1A1) was significantly higher in the SLEO group than that in the other groups (Figure 4d,e). Consistent with the previous results, the lipid transporter proteins Apolipoprotein B (APOB), Fatty acid transport protein 4 (FATP4), and Fatty acid binding protein 1 (FABP1) were all differentially elevated in the jejunum and ileum of the ATB group compared with the CON group (Figure 4f–h). The expression levels of APOB, FATP4, and FABP1 in the jejunum and ileum of the ATB+SLEO and SLEO groups were lower than those in the ATB group to varying degrees. Taken together, these results demonstrated that SLEO modulates both digestive processes and systemic lipid metabolism.

### 3.5. Effect of SLEO on the Intestinal Barrier of Silky Fowls

**Figure 5:**
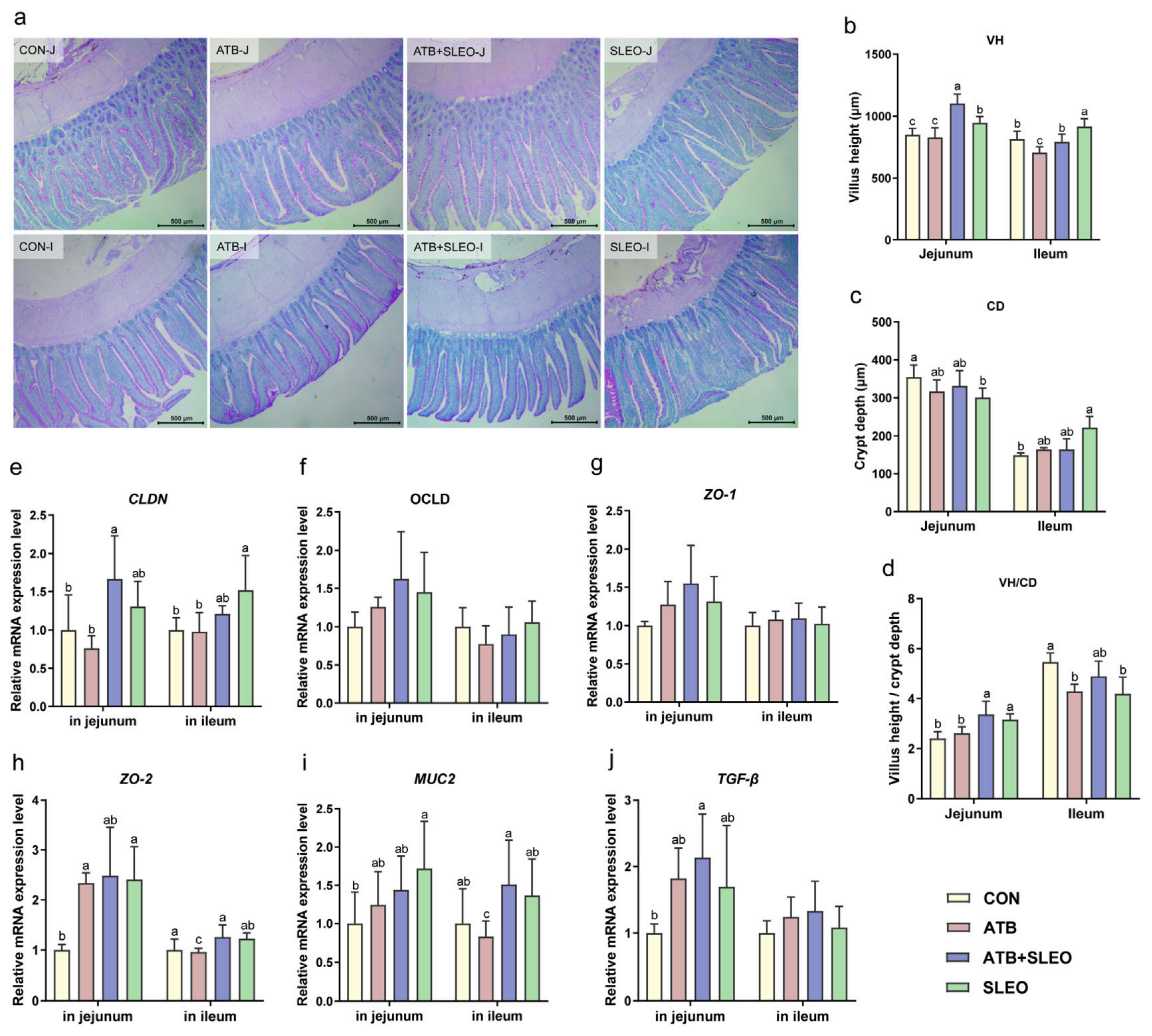
Effect of SLEO on the intestinal barrier of Silky fowls supplemented with antibiotics. (**a**) Microvillus morphology of jejunum and ileum. Scale bar: 500 μm; (**b**) VH; (**c**) CD, (**d**) VH/CD; The relative mRNA expression of (**e**) CLDN, (**f**) OCLD, (**g**) ZO-1, (**h**) ZO-2, (**i**) MUC2 and (**j**) TGF-β in the jejunum and ileum of Silky fowls supplemented with antibiotics. Different letters indicate significant differences between groups (*p* < 0.05).

To clarify whether SLEO optimizes growth performance by improving gut health in Silky fowls, firstly, we explored the effects of ATB and SLEO on the gut barrier. By observing the histological morphology of the jejunum and ileum, we found that ATB significantly reduced VH in the ileum compared to the CON group (Figure 5a,b). VH of the jejunum and ileum was significantly higher in the ATB+SLEO and SLEO groups compared to the ATB group. In addition, the CD of the jejunum was significantly shallower in the SLEO group versus the CON group, but the opposite was true in the ileum (Figure 5c). In the jejunum, VH/CD was markedly higher in both the ATB+SLEO and SLEO groups compared to the CON and ATB groups (Figure 5d). However, in the ileum, the VH/CD of the CON group was the highest among all groups.

Next, we took a more in-depth look at barrier gene expression in the small intestine of Silky fowls. Compared with the CON group, the relative mRNA expression of jejunal Claudin (CLDN), ileal Mucin 2 (MUC2), and Zonula Occludens-2 (ZO-2) was decreased, but jejunal ZO-2 was increased in the ATB group (Figure 5e,i,h). In addition, the relative mRNA expression of jejunal CLDN and Transforming Growth Factor-β (TGF-β) and ileal ZO-2 was increased in the ATB+SLEO group, as well as jejunal ZO-2 and ileal CLDN in the SLEO group (Figure 5e,j,h). Compared with the ATB group, jejunal CLDN and ileal ZO-2 and MUC2 were elevated in the ATB+SLEO group, as well as ileal CLDN, ZO-2, and MUC2 in the SLEO group (Figure 5e,h,i). Overall, the ATB+SLEO and SLEO groups exerted more positive effects on jejunal villus morphology and differentially influenced the expression of small intestinal barrier-related genes.

### 3.6. Effect of SLEO on the Diversity of the Cecal Microbiota in Silky Fowls

**Figure 6:**
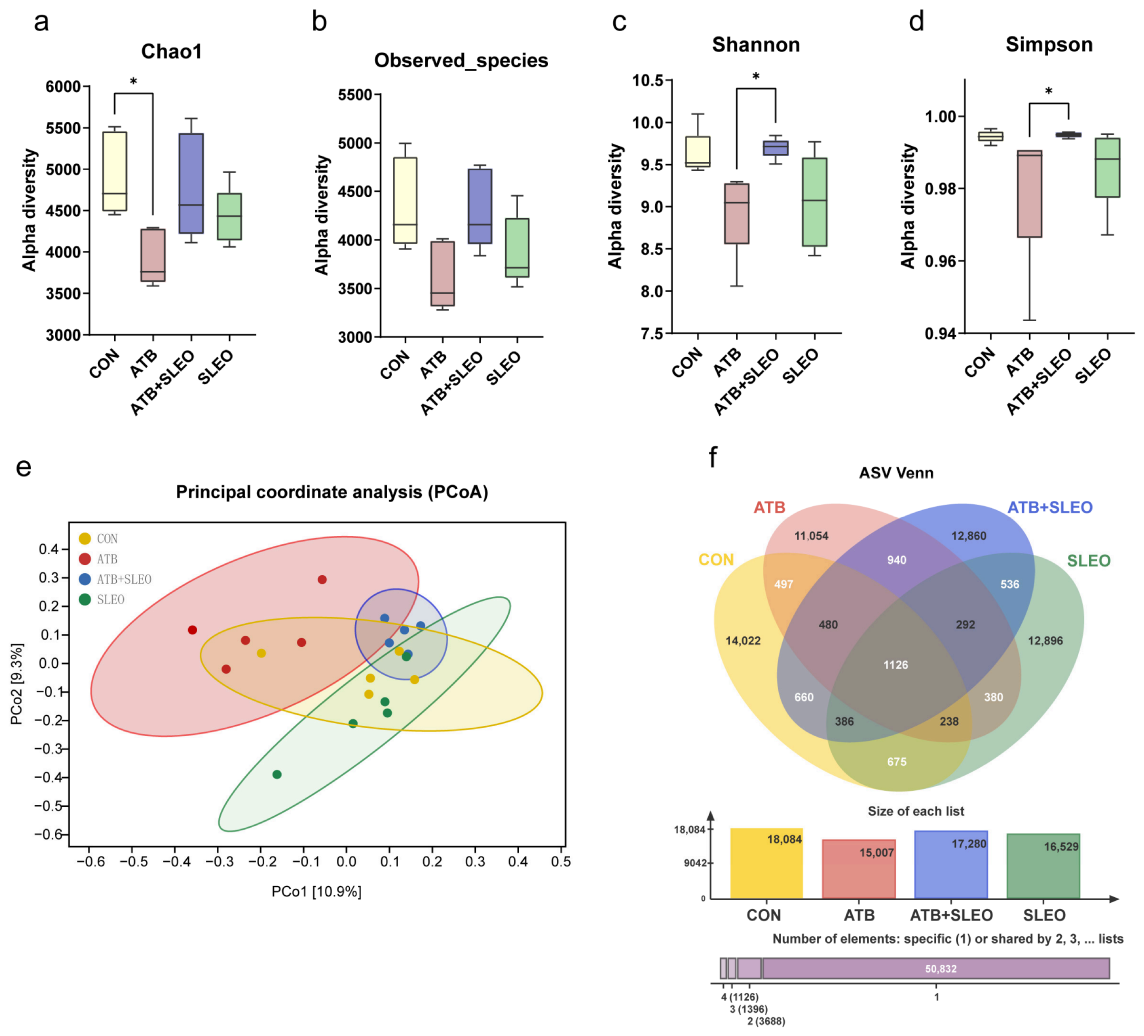
Effect of SLEO on the α and β diversity of gut microbiota in Silky fowls supplemented with antibiotics. (**a**–**d**) Alpha diversity of the cecum microbiota, including Chao1, Observed_species, Shannon and Simpson indices; (**e**) principal coordinate analysis (PCoA); (**f**) Venn of the cecum microbiota ASVs. * *p* < 0.05.

Disruption of the intestinal microbiota is a key characteristic associated with disease pathogenesis. Moreover, the long-term administration of low-dose antibiotics exacerbates this microbial dysbiosis [25]. Consequently, alterations in the gut microbiota were investigated in the present study. The Chao1 index was markedly reduced in the ATB group compared to CON (Figure 6a). Furthermore, SLEO obviously restored the Shannon and Simpson indices of the gut microbiota following ATB-induced disruption (Figure 6c,d). The Observed_species index showed a consistent trend of change across all groups, but the differences were not significant (Figure 6b). Principal coordinate analysis (PCoA) was performed to explore the differences in β-diversity among the groups (Figure 6e). PCoA revealed a more dispersed distribution of individual samples in the ATB group, indicating greater inter-individual variation in microbiota composition among ATB-treated Silky fowls. In contrast, samples in the ATB+SLEO group clustered more closely, suggesting higher homogeneity in microbial structure. The 95% confidence ellipses of the ATB and SLEO groups showed no overlap, confirming a significant separation between their microbiota profiles. The Venn diagram further demonstrated that the ATB group contained the lowest total number of Amplicon Sequence Variants (ASVs), as well as the fewest unique ASVs (Figure 6f).

### 3.7. Effect of SLEO on Differential Signature Bacterial Taxa in the Cecal Microbiota of Silky Fowls

**Figure 7:**
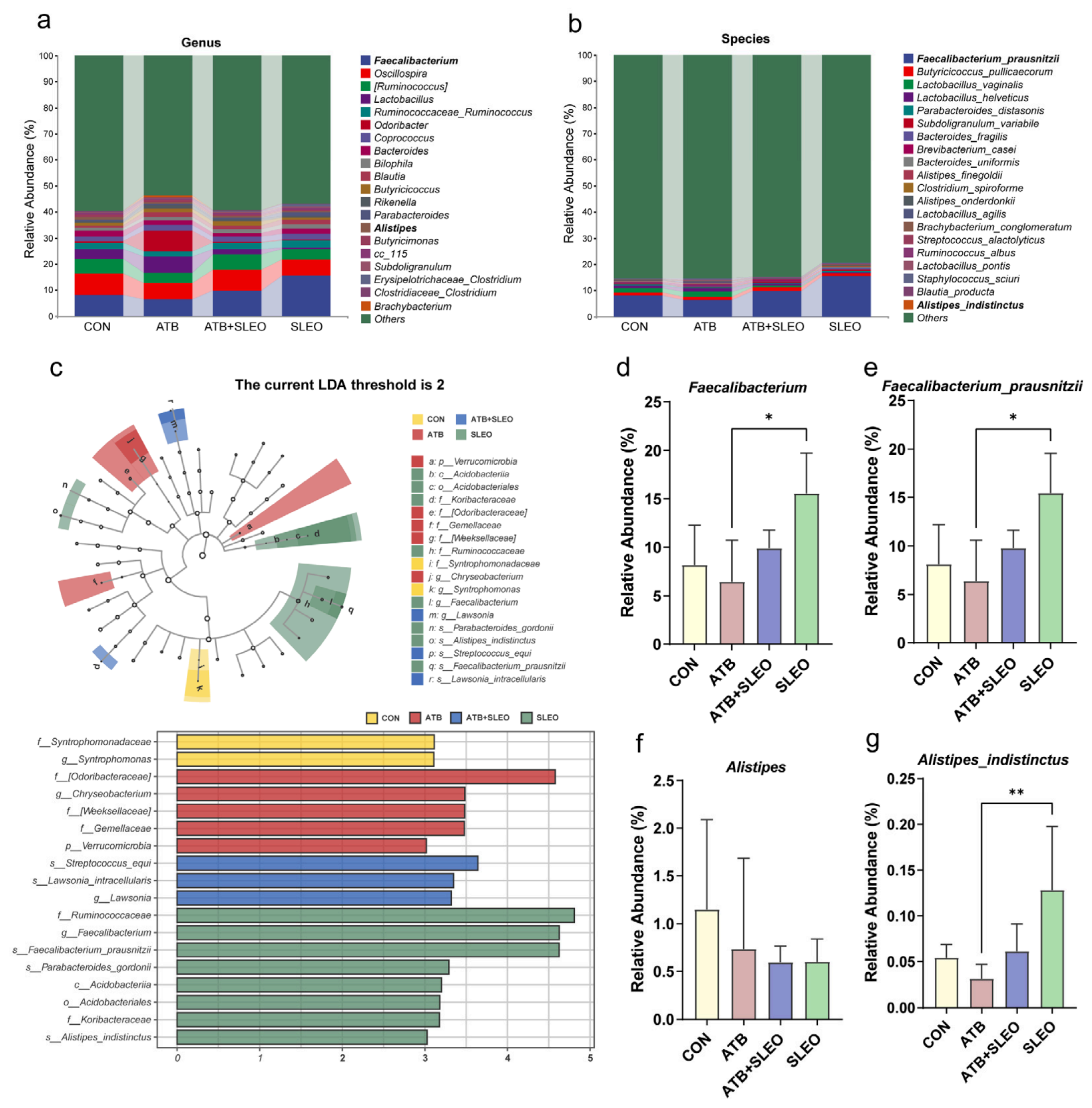
Effect of SLEO on the key species of gut microbiota in Silky fowls supplemented with antibiotics. (**a**,**b**) The relative abundance of cecum microbiota at genus-level and species-level; (**c**) LEfSe analysis and branching diagram of evolution scatterplot; (**d**) the relative abundance of *Faecalibacterium* and (**e**) *Faecalibacterium_prausnitzii*. The relative abundance of (**f**) Alistipes and (**g**) Alistipes_indistinctus. The R-value was screened with a value of 0.6. * *p* < 0.05, ** *p* < 0.01.

At the genus level, analysis of gut microbiota composition revealed that *Faecalibacterium, Oscillospira, and Ruminococcus* were the dominant genera (Figure 7a). At the species level, *Faecalibacterium prausnitzii* (*F. prausnitzii*) and *Butyricicoccus pullicaecorum* were identified as the dominant species (Figure 7b). Based on linear discriminant analysis effect size (LEfSe) analysis, 2, 5, 3, and 8 biomarker taxa were identified in the CON, ATB, ATB+SLEO, and SLEO groups, respectively (Figure 7c). In addition, among the top genera and species, the relative abundance of *Faecalibacterium* at the genus level, *F. prausnitzii* and *Alistipes indistinctus (A. indistinctus)* at the species level was higher in the SLEO group than that of the ATB group, and there was also a trend towards up-regulation in the ATB+SLEO group (Figure 7d,e,g).

### 3.8. Functional Units and Correlation Analysis of the Cecal Microbiota

**Figure 8:**
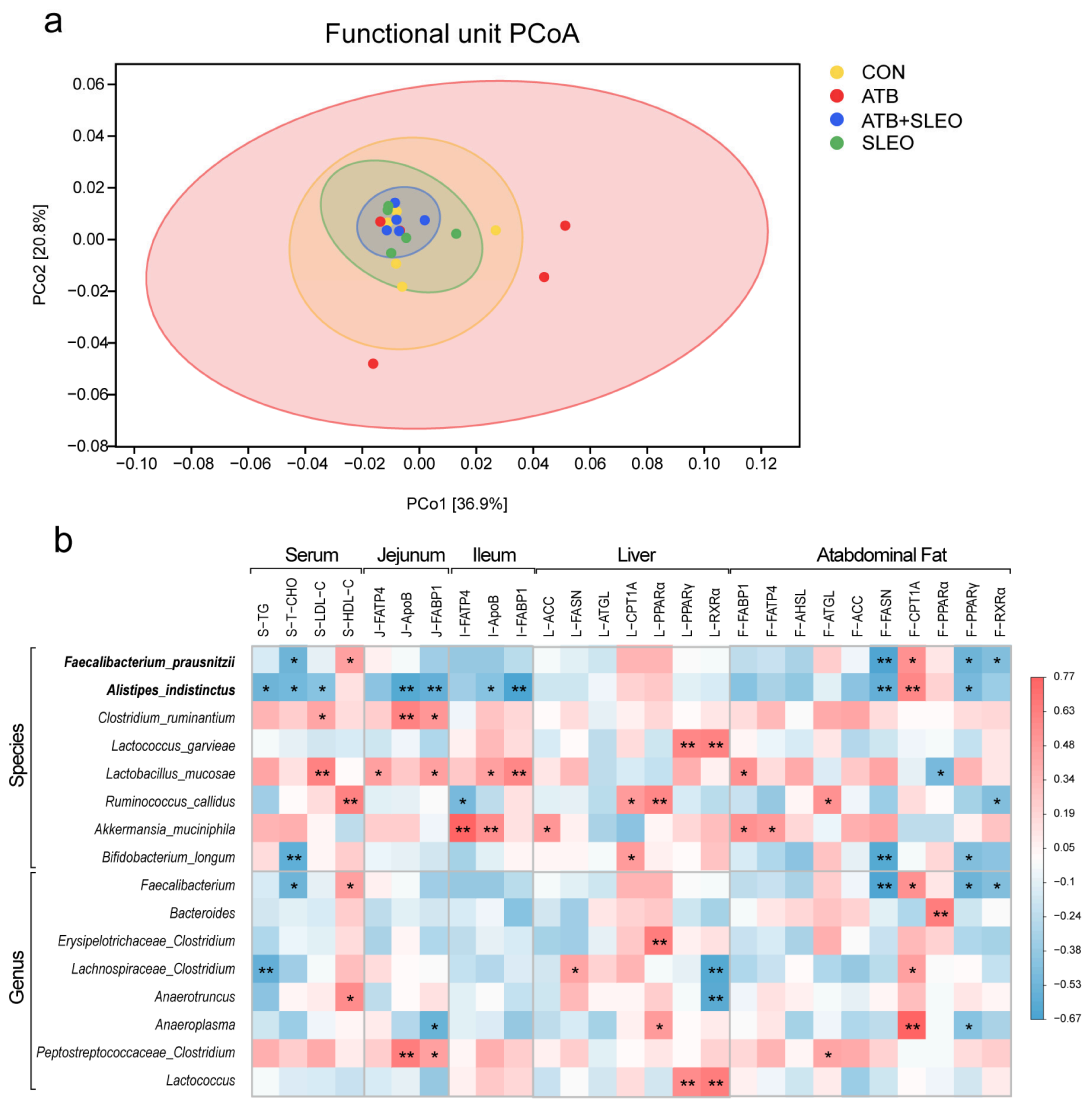
The function and correlation analysis of gut microbiota in Silky fowls supplemented with antibiotics. (**a**) The functional unit PCoA analysis of gut microbiota; (**b**) Spearman’s correlation analysis between cecum microbiota and lipid metabolism indicators. * *p* < 0.05, ** *p* < 0.01.

Given the vast number of functional units, we also performed PCoA to evaluate the similarities and differences in microbial community functional structure. The PCoA results revealed that ATB intervention significantly increased inter-individual variation in microbial community function, whereas ATB+SLEO reduced functional heterogeneity (Figure 8a). Moreover, a significant difference in functional structure was observed between the ATB and SLEO groups. Spearman correlation analysis between the top 50 microorganisms (at genus and species levels) and lipid metabolism-related indicators identified 8 genera and 8 species that exhibited strong correlations, respectively (Figure 8b). Notably correlated species included *F. prausnitzii*, *A. indistinctus*, *Clostridium ruminantium*, *Lactococcus garvieae*, *Lactobacillus mucosae*, *Ruminococcus callidus*, *Akkermansia muciniphila*, and *Bifidobacterium longum*. Of these, *F. prausnitzii* showed positive correlations with serum HDL-C and adipose tissue CPT1A but a negative correlation with serum T-CHO, adipose FASN, PPARγ, and RXRα. A. indistinctus was negatively correlated with serum TG, T-CHO, and LDL-C, as well as with small intestinal APoB and FABP1, and adipose FASN and PPARγ, while it was notably positively correlated with adipose CPT1A. Thus, we propose that SLEO may modulate lipid metabolism in Silky fowls primarily by enriching *F. prausnitzii* and *A. indistinctus*.

## 4. Discussion

Agricultural animals are often raised at high densities and rely on the use of antibiotics to promote growth and prevent disease [26]. As one of the “hardest hit areas of antibiotic use in animal agriculture”, the number of drug-resistant bacteria in chickens and pigs in China has almost tripled in the last 20 years [27]. The current development of antimicrobial resistance, which threatens our future, urges us to seek antibiotic-free health additives that are better suited to commercial animal farming. Instead of focusing on just one of these additives, by using a combination of feed additives, such as TCM, probiotics, and prebiotics, it is possible to effectively improve poultry products to help develop a sustainable poultry industry in the future [28,29]. However, essential oils, a key class of active constituents in TCM, suffer from an inherent limitation: high volatility during processing, comminution, and decoction, which hinders the full expression of their bioactivity. Therefore, building on our previous findings, the present study employed a modified SLBZS supplemented with its corresponding essential oils in order to comprehensively investigate the ability of TCM to enhance growth performance, reduce fat deposition, and improve intestinal health [10,16]. This study not only compared the effects of antibiotics and SLEO, but also evaluated the impact of SLEO on the adverse effects induced by antibiotics.

Excessive abdominal fat deposition impairs chicken health, compromises meat quality, and diminishes medicinal value, while prolonged antibiotic use may exacerbate this adverse fat accumulation. Consistent with previous reports on plant extracts reducing fat deposition, the present study found that SLEO similarly and significantly reduced abdominal fat deposition in Silky fowls [30]. SLEO also did not increase the accumulation of lipid droplets in the abdomen and liver, a negative effect associated with ATB administration. These alterations can lead to dyslipidemia. Subsequent findings revealed that ATB significantly elevated the levels of TG and T-CHO, an effect that was effectively reversed by SLEO. A review suggests that abnormal dynamic changes in TG and T-CHO, and increased fatty acid and sterol synthesis may contribute to the development of primary hepatocellular carcinoma [31]. Although SLEO was less effective than antibiotics in promoting body weight gain, it significantly improved the survival rate of Silky fowls, which may be associated with the fat-promoting effects of ATB. More importantly, SLEO exhibited positive effects in improving both reproductive and digestive functions, a benefit not observed with antibiotic administration.

Fat deposition is a complex process involving lipid metabolism, nutrient digestion, absorption, and transport, as well as intestinal health. Fatty acids are the body’s main source of energy [31]. FATP4, one of the major fatty acid transporters, is expressed at high levels in the apical part of mature enterocytes of the small intestine, and it promotes the absorption of long-chain fatty acids [32]. Once in the small intestinal enterocytes, FABP delivers fatty acids in a targeted manner to specific metabolic sites [33]. APoB-containing lipoproteins are assembled mainly in enterocytes and hepatocytes and are responsible for transporting food and endogenous fats to different tissues [34]. Since lipase was increased by SLEO intervention, we wondered if the expression of functional proteins responsible for transporting lipids was also changed. Apparently, SLEO affected the expression of APOB, FATP4, and FABP1 in the small intestine, as well as the expression of FATP4 and FABP1 in fat, which were upregulated by ATB. It is reasonable to believe that SLEO modulates lipid digestion and transport in Silky fowls. In the fed state, glycolytic products are used to synthesize fatty acids through neolipogenesis and combine with triacylglycerols, phospholipids, and/or cholesterol esters to form complex lipids [35]. HSL is a key enzyme in the regulation of lipids, highly expressed in adipose tissue, and catalyzes the breakdown of triacylglycerols and diacylglycerols [36]. HSL is responsible for mobilizing free fatty acids from adipose tissue [37]. In the adipose tissue of Silky fowls, the expression of HSL was increased by ATB and decreased by SLEO. In addition, SLEO was able to reverse the ATB-induced elevation of the key genes involved in adipogenesis, FASN and ACC, as well as the decrease in the fatty acid β-oxidation gene, CPT1A. Previous studies have suggested that the PPAR signaling pathway is closely related to glycolipid metabolism [38,39]. Saponins, the main components of many botanicals such as ginseng, frangipani, and licorice, can intervene in metabolic syndrome by targeting PPAR signaling pathways and adipokine levels, among others [40]. Flavonoids antagonize PPARγ, attenuate body weight gain, reduce white fat accumulation and hepatic steatosis, and improve glucose homeostasis in high-fat diet mice [41]. As a target gene of the PPAR family of nuclear receptors, PPAR/CPT1A not only mediates the fatty acid oxidation program, but also enhances mouse intestinal stem cell function and maintains intestinal homeostasis (Mihaylova et al., 2018) [42]. In our study, SLEO also seems to show potential to regulate PPARα, PPARγ, and RXRα. Solute carrier (SLC) transporters transport extracellular nutrients into the cell or shuttle across organelles to deliver intracellular nutrients [43]. The major pathways for the transport of dietary glucose from the intestinal lumen to enterocytes are SLC5A1 (SGLT1) and SLC2A2 (GLUT2) [44]. However, neither SLEO nor ATB appeared to affect glucose transport. The relative mRNA expression of the amino acid transporter y+LAT1 and glutamate transporter SLC1A1 was regulated after SLEO intervention, but not the oligopeptide transporter PEPT1. However, these negative changes related to lipid metabolism were observed in Silky fowls despite only early intervention with ATB. In contrast, even long-term SLEO interventions do not lead to disruption of lipid metabolism and can even eliminate the negative effects of early ATB. Collectively, SLEO systematically improves lipid digestion, transport, synthesis, and catabolism in the gut, liver, and adipose tissue.

The healthy growth of Silky fowls is not possible without the protection of the intestinal barrier. The intestinal epithelium constitutes a barrier separating the host from the food and microbiota in the gut, which plays an important role in the maintenance of intestinal homeostasis and the normal functioning of digestion and absorption [45]. The villi consist of terminally differentiated cells, including absorptive enterocytes, goblet cells, and enteroendocrine cells, whereas the crypts comprise stem cells and transit-amplifying cells [46,47]. Intestinal villi play an important role in nutrient absorption and maintenance of immune homeostasis in the animal organism. Therefore, we observed the morphology of villi in the small intestine of Silky fowls, and the results showed that ATB decreased the villus height of the ileum. While, SLEO had a positive effect on small intestinal villi morphology with or without the early application of ATB. The intestinal barrier is a complex structure that facilitates the absorption of nutrients as well as ensuring protection against intestinal pathogens and balancing homeostasis [48]. The proper function and regulation of the tight junction (TJ) of the intestinal barrier determines the ability of the epithelium to block solute and water leakage from the paracellular space and to function effectively as a transporter [47]. The ZO family of proteins is critical in TJ assembly, forming phase-separated droplets that recruit a variety of TJ components, including CLDN and OCLN [49]. Mucins are the main constituents of intestinal mucus, which has the dual property of protecting the inner surface of the mucosa and removing invaders [50]. TGF-β, more familiarly known to be associated with the regulation of intestinal immune responsiveness, has also been implicated in the expression of tight junction proteins and the maintenance of epithelial barrier function [51]. We assayed the mRNA expression of these TJs-related genes and found that in Silky fowls, the expression of ZO-2, CLDN, MUC2, and TGF-β was all increased by SLEO intervention. Previous studies have also confirmed that SLBZS promotes intestinal MUC2 secretion and increases the expression levels of ZO-1, CLDN, and OCLN, which enhances intestinal barrier function and restores homeostasis of the intestinal environment in mice with colitis, similar to our findings [52]. Our results suggest that the modulation of the intestinal barrier by SLEO may be one of the reasons for its growth-promoting properties.

For most herbivorous avian species, the cecum is an essential fermentation site, providing energy in the form of short-chain fatty acids (SCFAs), and essential nutrients such as vitamins and amino acids [53]. Gut microbiota metabolize products such as bile acids and SCFAs, and in this way influence lipid absorption and clearance, and regulate lipid metabolism and body weight by modulating energy extraction, processing, and storage [54]. Our study showed that microbial diversity and ASVs of Silky fowls were reduced by day 75 of age despite their exposure to antibiotics only during the first 10 days of growth. However, SLEO repaired microbial α-diversity and β-diversity disrupted by ATB, as well as increased ASV numbers. *F. prausnitzii*, the most abundant species across all groups of 75-day-old Silky fowls in our study, has been widely reported for its remarkable role in regulating lipid metabolism. *F. prausnitzii*, a health-associated butyrate-producing bacterium whose abundance is often reduced in patients with metabolic disorders and inflammatory bowel disease, is regarded as a key next-generation probiotic candidate with substantial therapeutic potential [55,56]. Reportedly, the Mediterranean diet (a plant-based diet) leads to multiple changes in the microbiome and metabolome and lowers plasma cholesterol in obese subjects, including the notable change in increasing *F. prausnitzii*, which degrades dietary fiber [57]. *A. Indistinctus* was identified as one of the species exhibiting the most pronounced differences from the ATB group following SLEO intervention. Several studies have shown that the presence of the genus *Alistipes* is associated with health-promoting phenotypes [58]. A study analyzing the gut microbiota and insulin resistance (IR) discovered that *A. indistinctus* preferentially consumes a wide range of carbohydrates while decreasing intestinal monosaccharide levels and improving lipid accumulation, thereby improving IR [59]. *Alistipes* had a hepatoprotective effect on the liver, and significant changes in *A. indistinctus* were also observed in type 2 diabetic rats ameliorated by the traditional Chinese herbal formula PuRenDan [60]. This implies that *F. prausnitzii* and *A. indistinctus*, as butyric acid-producing bacterium has a prominent role in regulating glycolipid metabolism.

Consistent with previous studies, the chicken cecum microbiota was significantly correlated with lipogenic and lipolytic metabolic genes [61]. Given the complexity of microbial interactions, the precise mechanism by which herbal compounds modulate the gut flora remains an open question. In this study, we focused on the observation that SLEO upregulated two bacterial species, *F. prausnitzii* and *A. indistinctus*, both of which have been independently linked to beneficial effects on lipid metabolism. Subsequent correlation analysis revealed strong associations between the abundance of these two species in the cecum and key serum lipid parameters as well as the expression of lipid metabolism-related genes in Silky fowls. Taken together, this study confirms that SLEO not only improves the growth performance, regulates lipid homeostasis, and modulates the gut microbiota in Silky fowls, but also significantly alleviates the metabolic side effects induced by ATB intervention. This finding provides evidence that the cecal microbiota may be involved in regulating host lipid homeostasis.

## 5. Conclusions

This study comprehensively compared the effects of SLEO and ATB across multiple dimensions. These results demonstrated that SLEO may not only regulate lipid metabolism balance and improve intestinal health in Silky fowls, including enhanced digestion, optimized nutrient transport, and strengthened intestinal barrier function, but also effectively prevent or reverse the adverse effects induced by ATB intervention. This research provides important evidence for clinical practices aimed at healthy, antibiotic-free poultry farming.

## Data Availability

The raw fastq files of 16S rRNA sequencing analysis in this study were deposited in the National Center for Biotechnology Information (NCBI BioProject: PRJNA1070841). All data sets generated or analyzed during this study are included in the article and Supplementary Materials. Data are available upon reasonable request.

## Supplementary Materials

The following supporting information can be downloaded at https://www.mdpi.com/article/10.3390/ani16152333/s1, Table S1: The nutritive composition of SLEO mixtures; Table S2: The ingredient composition of the basal diet (% on a fed basis); Table S3: AB-PAS staining procedure for intestinal sections; Table S4: Primers used for RT-qPCR.
