# Supplementary material for: Modified Shen Ling Bai Zhu San Combined with Its Essential Oil Regulates Growth Performance, Lipid Metabolism and Intestinal Health in Silky Fowls
Source: Animals (Basel). 2026 Jul 30;16(15):2333. doi: 10.3390/ani16152333 (PMC13463714; doi:10.3390/ani16152333)
Supplement: Supplementary file 1 [file animals-16-02333-s001.zip › animals-4429463-supplementary.pdf]

Supplementary Materials

Table S1. The nutritive composition of SLEO mixtures.

Nutritive ingredients	Content（%）	Inspection Basis/Test Methods
Crude fiber	15.40 %	GB/T 6434-2006
Crude protein	8.93 %	GB/T 6437-2018
Crude fat	2.70 %	GB/T 6438-2007
Ash	6.50 %	GB/T 6433-2006
Ca	0.64 %	GB/T 6432-2018
P	0.21 %	GB/T 6436-2018
Total polysaccharides	88.3 mg/g	Phenol-sulfuric acid method
Total polyphenols	5.67 mg/g	Colorimetric method
Flavonoids	4.78 mg/g	Spectrophotometric method

Table S2. The ingredient composition of the basal diet (% on a fed basis)

Ingredients (%)	Content		
	Day l~25	Day 26~50	Day 51~75
Corn	61.12	71.89	74.83
Canola meal	8.00	8.00	8.00
Soybean meal	18.76	6.90	3.17
Corn protein powder	4.00	4.00	4.00
Sunflower meal	3.00	3.00	3.00
Grade IV soybean oil	1.41	1.81	2.79
Talcum powder	1.47	1.28	1.27
Lysine sulfate	0.44	0.84	0.84
Calcium biphosphate	0.91	0.73	0.57
Sodium chloride	0.33	0.33	0.33
Argnine	0.01	0.28	0.30
DL-Methionine	0.07	0.23	0.16
Threonine	0.02	0.16	0.17
Valine	0	0.09	0.08
Choline chloride	0.08	0.05	0.08
Tryptophan	0	0.03	0.01
Premixes*	0.40	0.40	0.40
Total	100	100	100

*The premix per kilogram of feed contains (1) Day l~25: vitamin A 6,000 IU, vitamin B1 1.2 mg, vitamin B6 2.8 mg, vitamin B12 0.012 mg, vitamin D 1,200 IU, vitamin E 16.0 mg, vitamin K 1.2 mg, folic acid 0.6 mg, niacin 20.0 mg, D-Pantothenic acid 8.0 mg, biotin 0.06 mg, Cu 4.0 mg, Fe 32.0 mg, Mn 32.0 mg, Zn 20.0 mg, I 0.14 mg, Se 0.1 mg; (2) Day 26~50: vitamin A 5280 IU, vitamin B1 0.88 mg, vitamin B6 2.2 mg, vitamin B12 0.013mg, vitamin D 1 320 IU, vitamin E 13.2 mg, vitamin K 0.88 mg, folic acid 0.44 mg, niacin 17.6 mg, D-Pantothenic acid 6.6 mg, biotin 0.052 mg, Cu 3.52 mg, Fe 22.0 mg, Mn 26.4 mg, Zn 17.6 mg, I 0.154 mg, Se 0.066 mg; (3) Day 51~75: vitamin A 4,000 IU, vitamin B1 0.8 mg, vitamin B6 2.0 mg, vitamin B12 0.008mg, vitamin D 800 IU, vitamin E 12.0 mg, vitamin K 0.8 mg, folic acid 0.2 mg, niacin 14.0 mg, D-Pantothenic acid 6.0 mg, biotin 0.04mg, Cu 3.2 mg, Fe 20.0 mg, Mn 24.0 mg, Zn 16.0 mg, I 0.14 mg, Se 0.1 mg.

Table S3. AB-PAS staining procedure for intestinal sections.

Step	Procedure	Reagent/Solution	Time
1	Deparaffinization	Xylene	10 min × 2 times
2	Rehydration	Gradient ethanol (100%, 95%, 80%)	5 min each
3	Washing	Distilled water	3 min × 2 times
4	Alcian blue staining	Alcian blue solution (pH 2.5)	20min
5	Washing	Running tap water	5min
6	Oxidation	Periodic acid solution (0.5–1%)	10 min
7	Washing	Distilled water	3min
8	Schiff staining	Schiff's reagent	10min
9	Washing	Running warm water	10min
10	Counterstaining	Mayer's hematoxylin	1min
11	Washing	Running tap water	5 min
12	Dehydration	Gradient ethanol (80%, 95%, 100%)	3 min each
13	Clearing	Xylene	5 min × 2 times
14	Mounting	Neutral balsam	

Table S4. Primers used for RT-qPCR.

Gene	Primer sequence (5′→3′)		Accession number
ACTB	Forward:	TTGGGTATGGAGTCCTGTGGT	NM_205518.2
	Reverse:	AATGCCAGGGTACATTGTGGT	
CLDN	Forward:	ATGACCCTTTCACACCCGTT	NM_001013611.2
	Reverse:	GCTGGGTGGGTAGGATGTTT	
ZO-2	Forward:	GCAAGGTGAAGCAGTTTGGG	XM_046934796.1
	Reverse:	ATGCACCTCCCTCTCCATCT	
MUC2	Forward:	AACCACCACAAGCCAGTCTC	XM_040673077.2
	Reverse:	TCCAGGTGCATTCACAATTTCC	
TGF-β	Forward:	ATGTGTTCCGCTTTAACGTGTC	NM_205454.2
	Reverse:	GCTGCTTTGCTATATGCTCATC	
GLUT2	Forward:	TGTTAGCAGCACACCACACA	NM_207178.2
	Reverse:	CCCCTCTGCTCTTGTGAGAC	
SGLT1	Forward:	TGTCTCTCTGGCAAGAACATGTC	XM_046928028.1
	Reverse:	GGGCAAGAGCTTCAGGTATCC	
PEPT1	Forward:	CTCCAGGCTGCTAAAAGTGG	XM_046906441.1
	Reverse:	AACTGGGTGGGCTTTAAGGT	
y+LAT1	Forward:	CAGAAAACCTCAGAGCTCCCTTT	XM_046911929.1
	Reverse:	TGAGTACAGAGCCAGCGCAAT	
SLC1A1	Forward:	CTGGGAAGATTGGTTTGCGAG	XM_046936555.1
	Reverse:	GTTTTGTTCTCCAAAACTCCTGC	
APOB	Forward:	CCTGCCATGGGAAACATTAC	NM_001044633.2
	Reverse:	TGCAGTGCATCAATGACAGA	
FABP1	Forward:	ACTGGCTCCAAAGAATGACCAATG	NM_204192.4
	Reverse:	TGTCTCCGTTGAGTTCGGTCAC	
FATP4	Forward:	ATACCTCTGGCACTACGGGAAT	XM_046929199.1
	Reverse:	CATACATCACATCATCGGGTCT	
HSL	Forward:	CTCTCCTCATTGGCTTCGGAC	XM_040695201.1
	Reverse:	AAAGGTGTTGGAGGCGTTGG	
ATGL	Forward:	AAGTCCTGCTGGTCCTCTCCTTG	EU240627.2

	Reverse:	AGTGTTGTCCTCCATCTGGTCCTC	
ACC	Forward:	CCGAGAACCCAAAACTACCAG	XM_046929960.1
	Reverse:	GCCAGCAGTCTGAGCCACTA	
FASN	Forward:	CTATCGACACAGCCTGCTCCT	NM_205155.4
	Reverse:	CAGAATGTTGACCCCTCCTACC	
CPT1A	Forward:	CGAGTCAGACACCACAGCAACAC	XM_046918285.1
	Reverse:	CACCGTAACCATCATCAGCCACAG	
PPARα	Forward:	CCTTTCACCAGCATCCAGTCCTTC	XM_046906400.1
	Reverse:	TGTACTCCGTAATGGTAGCCTGAGG	
PPARγ	Forward:	CAGTGGATCTGTCTGCGATG	XM_046925952.1
	Reverse:	CTTTGGCAATCCTGGAGCTTG	
RXRα	Forward:	GATGCGAGACATGCAGATG	XM_040686122.2
	Reverse:	GTCGGGGTATTTGTGCTTG	
